# Predicting pediatric anxiety from the temporal pole using neural responses to emotional faces

**DOI:** 10.1038/s41598-021-95987-4

**Published:** 2021-08-18

**Authors:** Jeffrey Sawalha, Muhammad Yousefnezhad, Alessandro M. Selvitella, Bo Cao, Andrew J. Greenshaw, Russell Greiner

**Affiliations:** 1 Department of Psychiatry, University of Alberta, Alberta, Canada; 2 Department of Computing Science, University of Alberta, Alberta, Canada; 3 Alberta Machine Intelligence Institute (Amii), Alberta, Canada; 4 Department of Mathematical Sciences, Purdue University, Fort Wayne, United States; 5 eScience Institute, University of Washington, Seattle, WA USA

**Keywords:** Cognitive neuroscience, Attention, Social behaviour, Diagnostic markers, Predictive markers

## Abstract

A prominent cognitive aspect of anxiety is dysregulation of emotional interpretation of facial expressions, associated with neural activity from the amygdala and prefrontal cortex. We report machine learning analysis of fMRI results supporting a key role for a third area, the temporal pole (TP) for childhood anxiety in this context. This finding is based on differential fMRI responses to emotional faces (angry versus fearful faces) in children with one or more of generalized anxiety, separation anxiety, and social phobia (n = 22) compared with matched controls (n = 23). In our machine learning (Adaptive Boosting) model, the right TP distinguished anxious from control children (accuracy = 81%). Involvement of the TP as significant for neurocognitive aspects of pediatric anxiety is a novel finding worthy of further investigation.

## Introduction

Clinical anxiety is associated with inability to control or auto-regulate one’s autonomic response^1^, and it is the most common mental illness among children and young adults^2^ with a lifetime prevalence rate of 28.8%^3,4^. The median age of onset for all anxiety disorders, at 11 years old, marks this as the earliest among all psychiatric disorders, and over 30% of pediatric cases meet criteria for two or more subtypes^2,4^. Despite high prevalence and possible early onset, these disorders are often under-reported because of conflation of normal developmental-behavioral patterns with anxiety symptoms. Assessment is typically limited to diagnostic interviews and questionnaires to produce a diagnostic label, which comes with its own validity issues^3,5,6^. Anxiety and related symptoms may have profound effects on neurological functioning in a child’s rapidly developing brain^5,7^ and, over extended periods of time, may lead to cognitive, social, and emotional deficits^1^. For example, adolescents with high trait anxiety exhibit an attentional bias (pay greater attention) to negatively valenced faces^8,9^. Although socioemotional circuits in the brain have been implicated in numerous psychiatric disorders, including anxiety^10^, such cognitive deficits have rarely been used as an indication of brain mechanisms underlying psychopathology.

Cognitive models of anxiety suggest that negative biases exist for performance on information-processing tasks^11^—in particular, anxious individuals allocate greater attention to negative or threatening stimuli^9^. They may find threatening words more salient, and may remember them more often than non-threatening words^12,13^. Emotional facial expressions are often perceived as more negative or threatening (even if they are typically judged as neutral), and this is associated with activation of affective brain circuits^8,14^. These attentional and perceptual biases are thought to be an important feature underlying the etiology of anxiety disorders, a view supported by functional neuroimaging studies. Despite the likely clinical significance of these biases, few studies have focused on the adolescent population during a facial emotional processing task^15–17^, and rarely have machine learning methods been applied to assess if neural signatures underlying such biases may be used to identify children suffering from anxiety.

Functional neuroimaging measurements during facial processing tasks have helped reveal neurological underpinnings of emotional regulation. Overall, there is evidence of dysregulated fear-circuitry related regions, including the amygdala and prefrontal cortex (PFC)^18^. Children with panic disorder (PD) or generalized anxiety disorder (GAD) may exhibit exaggerated amygdala responses to fearful faces compared to non-anxious or depressed children^19^. Hyperactivity has been observed in several limbic brain regions in separation anxiety disorder (SAD) patients when responding to fearful faces, including the fusiform gyrus (associated with facial recognition), and there is evidence of increased connectivity between the fusiform gyrus and amygdala, as well as the fusiform gyrus and the superior temporal sulcus^20^. Also, abnormal neural responses to emotional faces have been reported for adults with GAD, PD and SAD, with greater right amygdala activation reported in response to fearful versus happy faces^21^. From these studies, similar amygdala activation patterns to happy faces were reported for both patients and controls, indicating that this area is also responsive to positively valenced facial expressions. Increased responses in the superior temporal sulcus, an important area for deriving social and emotional information, were observed for SAD and PD patients viewing fearful faces. The findings mentioned above have focused mainly on between group differences or similarities. In recent years, advanced data analysis methods, such as machine learning, have enabled accurate prediction on an individual basis^22^. This approach holds the potential to enable improvement of clinical decision-making (such as diagnostic assessments), and to provide evidence-based determination of which brain regions display the largest differences between individuals in different classes (diagnosis versus no-diagnosis cases), based on fMRI data, while the participants perform passive or active tasks. In this study, we explore whether machine learning analysis of emotional facial recognition may allow us to identify, with higher precision, which children will suffer from anxiety.

**Figure 1:**
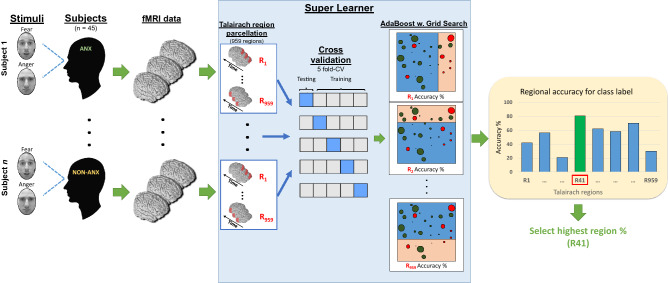
Processing pipeline for selecting the best Talairach region. Super learner (SL) parcellates the whole-brain data into 959 regions based on the Talairach atlas. It determines which region can best distinguish between anxious and non-anxious children. The SL uses a nested cross-validation process to hyper-tune parameters for an AdaBoost model. The SL uses the regions as a hyperparameter within this process. The region with the highest average accuracy was selected for our analysis. Note: each time point produced its own prediction; we labeled each person with the majority vote over the time points for that subject.

Conventional neuroimaging analysis of two different populations (anxious versus non-anxious) involves comparing neural activation of various regions between the groups, anticipating that comparing the blood oxygenation level-dependent response (BOLD) at specific voxels will show significant differences. However, this analysis mainly focuses on univariate and group-level statistics, and may not lead to predictions for individual cases due to the overlap of neural responses at any given voxel^22,23^. Multivoxel pattern classification (MVPA) applies machine learning algorithms to fMRI BOLD signals to produce predictive models^24^. These models can categorize brain patterns into distinct stimulus conditions (emotional faces) or groups based on spatial and temporal discriminative neural signatures from high dimensional neuroimaging data^23^. This analysis can also reveal which brain regions differ the most between two groups or stimulus conditions. Neural signatures can be further clarified with advanced alignment techniques like the probabilistic shared response model (SRM), which aligns patterns of neural responses across subjects into a common, lower-dimensional space^25^. Here, we demonstrate that MVPA can be used to decode brain patterns related to the disease state of adolescent children. MVPA may also indicate which brain regions are key aspects for altered functional connectivity in anxious children in this context.

Using a publicly available dataset (https://openneuro.org/datasets/ds000144) consisting of task-based fMRI data from children with anxiety disorders such as SAD, SP and GAD: (1) We applied a data driven approach to determine a combination of brain regions to distinguish anxious versus non-anxious children with above chance accuracy based on facial-emotional processing. (2) We examined neural correlates of angry and fearful faces to distinguish those stimuli using similar techniques. Figures 1 and 6 illustrate the analysis and posthoc pipeline for these research questions (refer to the online methods for a full description of the study).

Our approach is based on task-based fMRI data rather than resting-state MRI^26^. A key question is whether task-based fMRI derived regions can be linked to various resting-state networks in this context. Many reports indicate that a functional imbalance in large scale networks, such as the default mode network (DMN), the salience network, or the affective network, play a crucial role in anxiety disorders^27–29^. However, we found that intrinsic resting-state network activity may not differ significantly from task evoked responses, in accordance with several sources suggesting that task-based responses are related to modest changes compared to intrinsic activity^30,31^. If certain regions arise as significant predictors of childhood anxiety using machine learning analysis for the task-based approach, it will be important to compare them with components of resting-state networks previously associated with anxiety.

Research has not yet established a clear link between brain-behavioral function and clinical diagnosis in children, which is problematic. The hope is that research into developmental psychopathology will bridge the gap between psychiatric practice and neuroscience^32^. Our current approach may enable us to relate functional brain measures to pediatric diagnoses in anxiety disorders, and may also help to generate new therapeutic insights.

To date, we are not aware of published attempts to use machine learning to validate psychiatric disorders in young children (in this case, 5–10 years old) using task-based fMRI data. We propose that distinguishable neural substrates in anxious vs. non-anxious children can be identified with our machine learning approach for individual predictions on a case by case basis.

## Results

### Clinical and demographic statistical analysis

Table 1 shows demographic and clinical data from 22 anxious and 23 non-anxious children in our sample, including comorbidities and overlap between various anxiety disorders. When testing for group differences in age, a two-tailed t-test revealed a significant difference between the anxious and non-anxious group ($$t_{(43)} = 2.03, {p} < 0.05$$) and the SP cohort ($$t_{(32)} = 2.36, {p} < 0.02$$). When measuring functional impairment and emotional symptoms, the anxious groups differed significantly from the non-anxious group ($$ {p} < 0.005$$). No statistical differences were found when comparing sex, ethnicity, handedness, IQ, or socioeconomic status between any of the groups. Note, we compare non-anxious individuals against each of the anxious subtypes for this statistical test only. Our machine learning task combines all anxious subtypes into the main anxious group as shown in Table 1.

**Table 1:** A summary table of demographic and clinical symptom scores of participants. The anxious group contained individuals with one or more anxiety disorders from the mentioned subtypes. The 3 anxiety subtypes are not mutually exclusive. T-tests were conducted between the non-anxious group and all anxious subtypes, as well as the whole anxious group. Mean values and standard deviations are reported for all 4 groups. Significant difference from non-anxious children at $${}^{*}p~<~0.05$$, $${}^{**}p~<~0.005$$.

		Non-anxious (N=23)	Anxious (N=22)	Generalized anxiety (N=15)	Separation anxiety (N=10)	Social Phobia (N=11)
Demographics	Age at scan	7.48 (1.04)	6.86 $(0.99)^{*}$	6.86 (1.06)	7.00 (1.33)	6.63 $(0.81)^{*}$
	Female	13	16	12	7	8
	Ethnicity	12	10	8	6	3
	Below poverty	4	6	5	5	2
	Handedness (right)	16	18	14	7	8
	IQ	104.48 (14.02)	103.86 (10.81)	103.52 (11.51)	103.20 (10.63)	106.18 (9.54)
Symptoms	Impairment (0–10)	0.74 (1.09)	3.5 $$(2.35)^{**}$$	3.93 $$(2.66)^{**}$$	3.80 $$(2.62)^{**}$$	3.28 $$(1.68)^{**}$$
	Emotional symptoms (0–14)	2.17 (1.99)	6.54 $$(2.91)^{**}$$	7.26 $$(3.13)^{**}$$	8.40 $$(2.91)^{**}$$	5.81 $$(2.40)^{**}$$

### Anxious versus non-anxious classification analysis

#### Machine learning analysis

**Figure 2:**
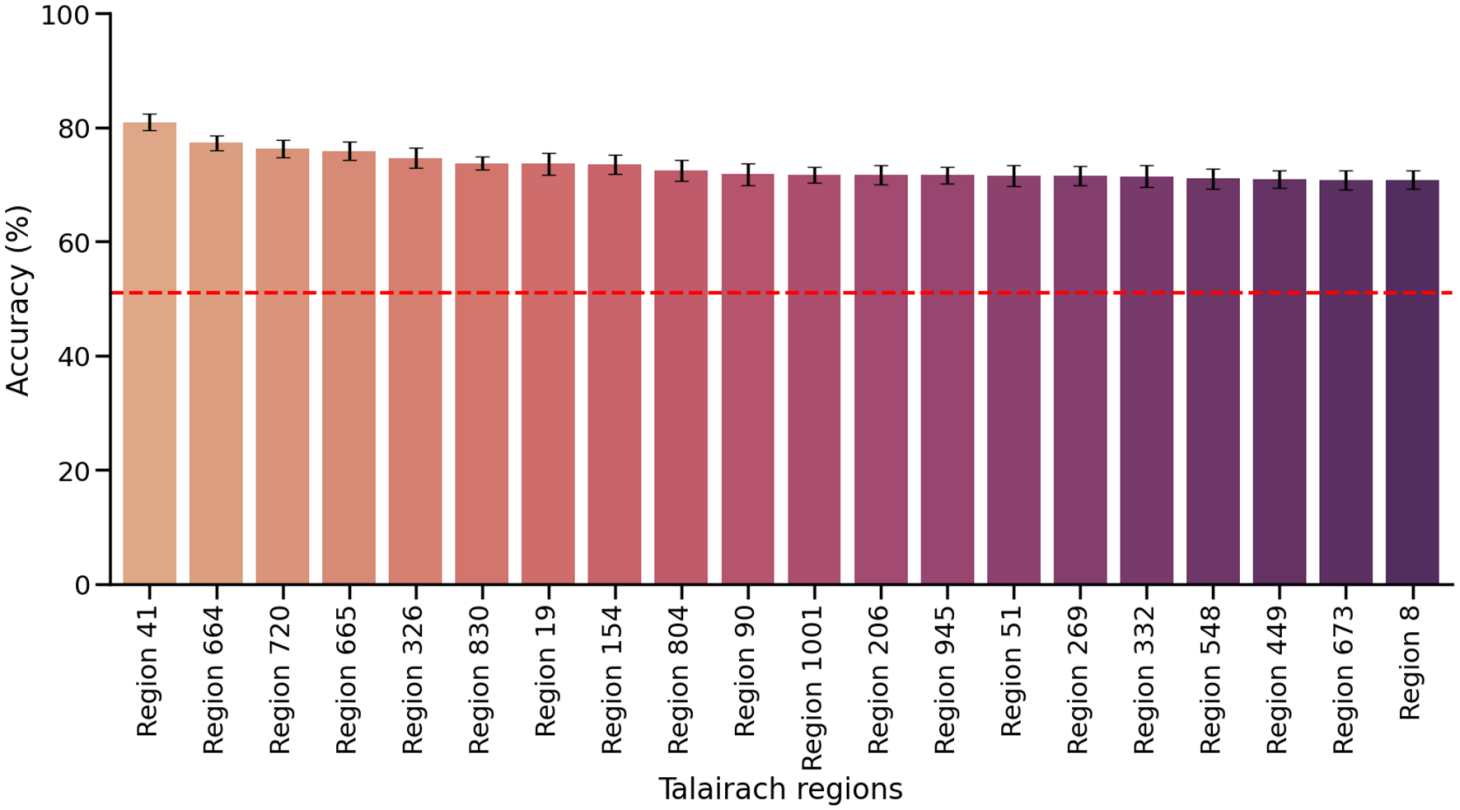
Top 20 Talairach regions by accuracy (%). The SL considered classifiers produced by base learners, each applied to a specific region. This shows the mean 5-fold internal CV accuracies, for the top 20 regions. The SL selected region # 41 to best distinguish anxious from non-anxious children. Other highly ranked regions include region # 664 (Right cerebrum, Limbic lobe. Posterior cingulate white matter) and region # 720 (Left Cerebrum, Transverse temporal gyrus, Brodmann area 38). All error bars represent the standard error of each participant across inner-CV folds from the SL. Red stripped line represents baseline accuracy for the majority group (51.1 %).

Using the Talairach atlas (2mm), we use a super learner (SL) to segment the whole-brain data into 959 regions, then train a AdaBoost with the regions serving as a hyper-parameter. Here, the SL used nested cross validation (CV) (5-CV on the outer and inner loop) to partition and fine tune hyper-parameters. Figure 1 illustrates the machine learning pipeline for this section. The SL achieved the highest accuracy by using voxels from region $\#41$ with 81% (STE ± 1.46%) (MNI: x = 40, y = 11, z = − 35) (Right temporal pole, right Cerebrum, Superior Temporal Gyrus, Brodmann area #38). When examining differences between the second and third ranked regions from our internal CV process, the accuracy of region $\#41$ was not statistically different than region $\# 664$, which had an accuracy of 77% (STE ± 1.33%) (MNI: x = 10, y = − 50, z = 20) (Right cerebrum, Limbic lobe. Posterior cingulate white matter) ($t_{(21)} = 0.16, p = 0.87$) or region $\#720$, which had an accuracy of 76% (STE ± 1.52%) (MNI: x = − 52, y = − 19, z = 7) (Left Cerebrum, Transverse temporal gyrus, Brodmann area 38) ($t_{(21)} = 0.18, p = 0.85$). This can be viewed in Fig. 2, which includes the top 20 ranked regions from the SL’s internal CV mean accuracy. Note that region $\#664$ included white matter tracts that were proximal and inside region #41, and region $\#720$ was the left hemisphere temporal pole.

Classification performance was measured using accuracy (percentage of correctly classified participants), precision, sensitivity, recall, and F1-score. Using our SL, we achieved an accuracy of 81% an overall precision of 80% as seen in Fig. 7A, recall at 80% and an F1-score of 80%. Table S1 reveals the detailed results of our final SL model.

**Figure 3:**
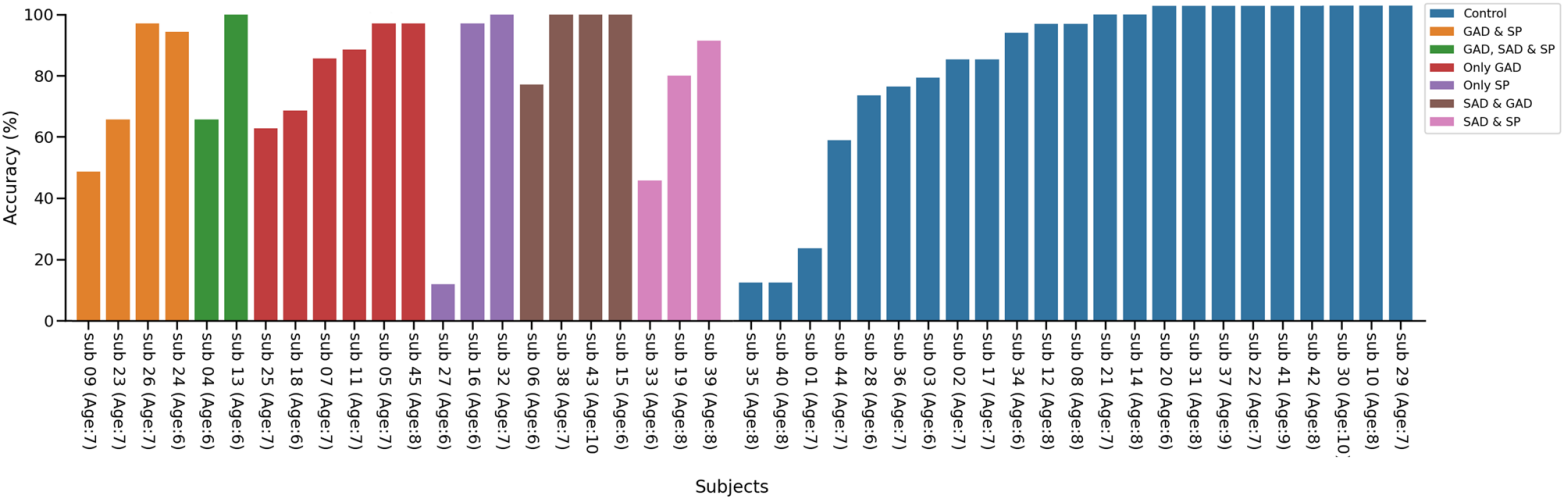
Individual accuracies (%) of participants based on sub-classes from final super learner model. A plot of each participants mean accuracy across 35 time points, grouped by subtypes from our final AdaBoost model, which learned from voxels in only region # 41.

**Figure 4:**
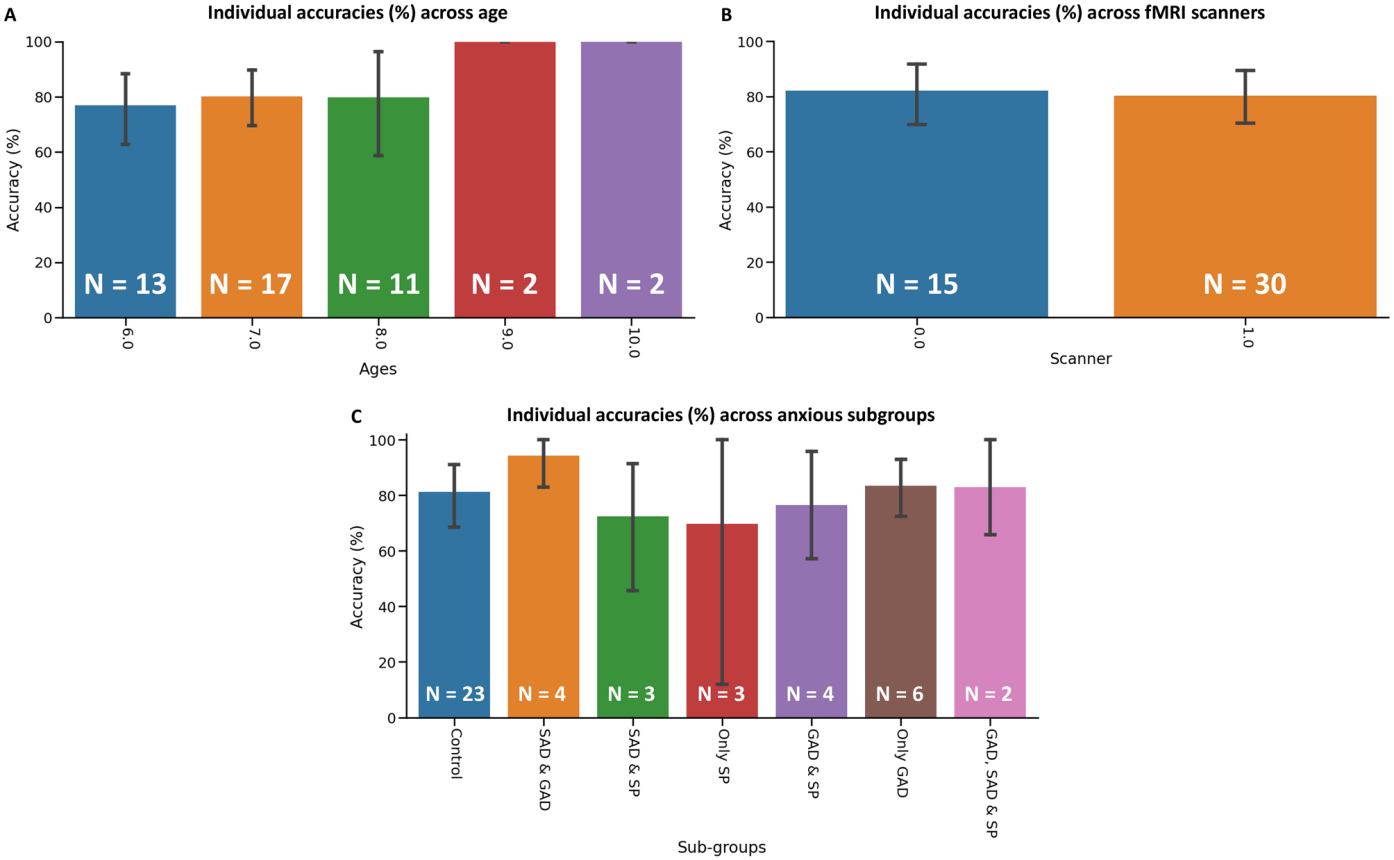
(**A**) Individual accuracies grouped across age (5–10) from super learner model. Mean accuracy of individuals grouped by age. (**B**) Individual accuracies grouped across fMRI scanner sites. Mean accuracy of individuals grouped by the two fMRI scanner sites. (**C**) Individual accuracies grouped by anxious subtypes. Mean accuracy of individuals with one or more comorbid anxiety diagnosis. All error bars represent the 95% confidence intervals through bootstrapping of individual accuracies.

To ensure these results were not driven by several confounding factors, we observed individual accuracies across a number of variables including age, anxious subtypes, and the different fMRI scanner sites. First, we plotted the individual accuracy of every participant based on their class label in Fig. 3. In Fig. 4C, we can see the average accuracy for each class subtype. The control group had an average accuracy of 81%. Our model returned an average accuracy of 76% for children with both GAD and SP, 76% for SP and SAD, 83% for GAD only, 70% for SP only, 94% for SAD and GAD, and 72% for SAD and SP. Of note, children with only SP (n = 3) had large variations between accuracies, so the 95% confidence interval (CI) is quite expansive. Figure 4A shows accuracies grouped by age. With region #41 alone, our AdaBoost model could perfectly predict children ages 9–10 while also maintaining accuracies above 75% for all other age groups. We also plotted accuracies of individuals based on scanner sites in Fig. 4B to determine whether a given scanner was driving our model results. Although scanner #0 had half the participants compared to scanner #1, the accuracies between the two are almost identical (scanner #0 = 82%, 95% CI (70.62–93.57), scanner #1 = 80%, 95% CI (70.24–90.45).

#### Statistical analysis of Talairach region #41

**Figure 5:**
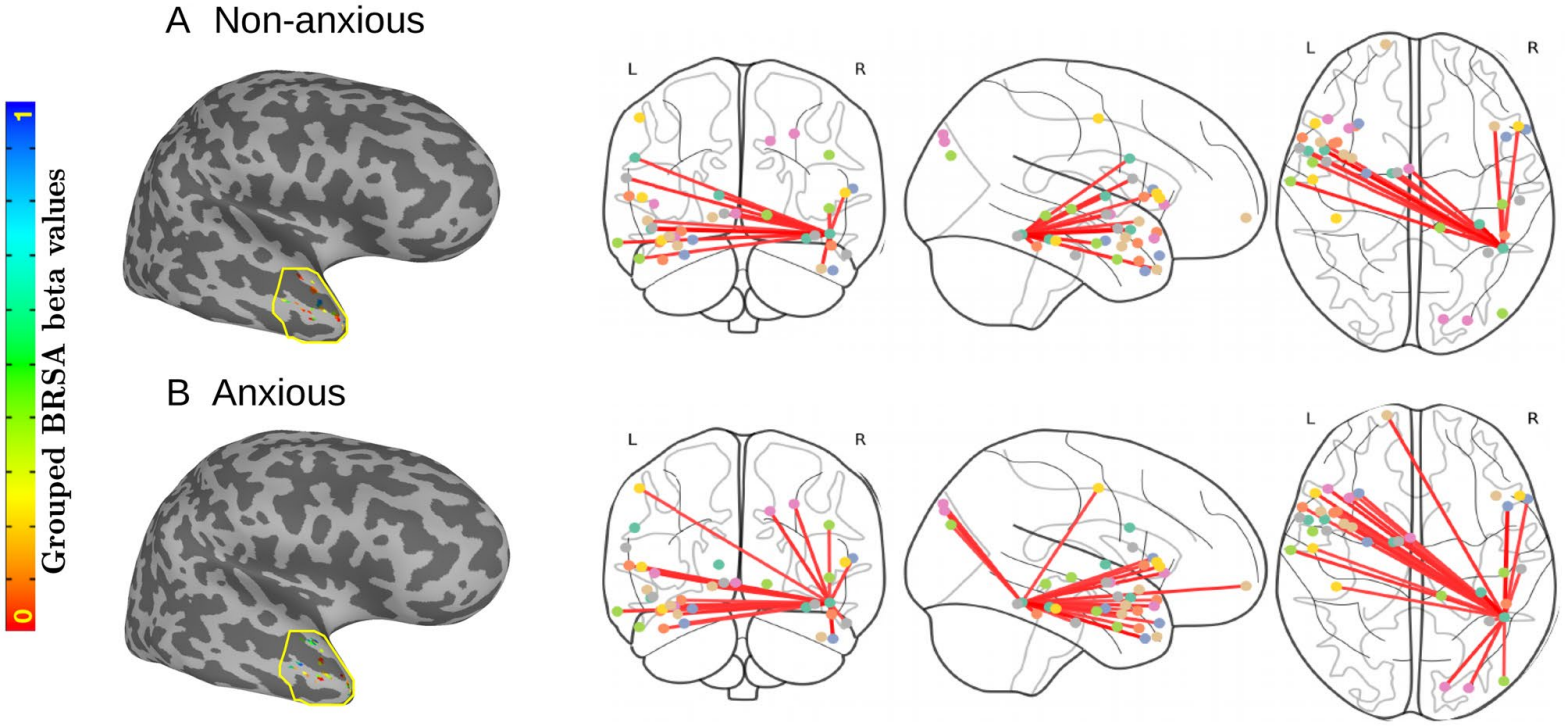
(Left) Grouped Bayesian representational similarity analysis of region #41. A between-group ROI analysis was used to examine activation differences for anxious versus non-anxious children. In our statistical comparisons, 685 pairs of mean beta values in region $\#41$ were compared between each group using a Mann-Whitney U-test (two-tailed). (U = 215017.00, $p < 0.005$). (Right) Fully connected network analysis of Talairach region #41 with anxious versus non-anxious children. A visual representation of regions connected with region $\#41$. Dots represent the 38 regions with absolute correlation thresholds greater than 0.6, each connected with a red line to region $\#41$. (**A**) Fully connected network for non-anxious children. (**B**) Fully connected network for anxious children.

Here, we conducted a high level, between-group ROI analysis for region $\#41$ to examine the neural response differences between anxious and non-anxious children. Using the neural responses from our second level grouped Bayesian representational similarity analysis (GBRSA), we compared activation in this region by using a mask to confine our analysis. Details on this procedure can be found in the online methods. This brain mask was used to extract this specific region only for our statistical test. Figure 5 (left) shows the region-based neural responses for both anxious and non-anxious children. We compared all 685 pairs of voxels in this region using a Mann-Whitney U test (two-tailed). The statistical test confirmed that the distribution of beta values from our GBRSA analysis for the anxious group was significantly different from the non-anxious group in region $\#41$ ($U=215017.00, p < 0.005$).

Figure 5 (right) represents the fully connected network analysis for region #41. We examined neural activity of highly correlated brain regions with region #41 in both groups. We only drew connected regions with an absolute correlation threshold value of 0.6 or higher, and represented those using the red lines extending from region #41. Table 2 reveals 26 regions that have a correlation value of greater than 0.60 in the anxious group, but only 16 for the non-anxious group.

**Table 2:** Talairach regions most correlated with region #41.

Brain regions correlated with Region #41	Non-anxious	Anxious
R. Temp. lobe, STG	0.87	0.90
L. STG, Brodmann 38	0.83	<0.60
R. ITG, Brodmann 21	0.65	<0.60
R. MTG, Brodmann 21	0.71	<0.60
L. STG	0.74	0.75
L. Fusiform Gyrus	<0.60	0.63
L. Fusiform Gyrus, Brodmann 20	<0.60	0.66
L. Fusiform Gyrus, WMT	<0.60	0.66
L. Parahippocampal Gyrus, Brodmann 36	<0.60	0.66
L. Fusiform Gyrus, Brodmann 36	<0.60	0.68
R. Amygdala	<0.60	0.67
L. Front. lobe, IFG	<0.60	0.67
R. Front. lobe, IFG	<0.60	0.83
R. IFG Brodmann 47	<0.60	0.72
R. Font. lobe, IFG, WMT	<0.60	0.74
R. Parahippocampal Gyrus, Brodmann 20	<0.60	0.69
R. Parahippocampal Gyrus, Brodmann 38	0.72	<0.60
R. Frontal lobe, STG	<0.60	0.72
R. Temp. lobe, IFG	0.73	0.71
L. Sub-Gyral	0.64	<0.60
R. Temp. Sub-Gyral Brodmann 13	0.66	<0.60
R. Temp. Sub-Gyral	0.73	<0.60
R. Front. Lobe, Sub-Gyral Brodmann 47	<0.60	0.63
R. Brainstem Extra-Nuclear WMT	<0.60	0.64
R. Cerebrum Extra-Nuclear WMT	<0.60	0.66
L. Temp. Lobe, Insula Brodmann 13	0.64	<0.60
L. Front. Lobe, IFG Brodmann 45	<0.60	0.73
R. Front. Lobe, IFG Brodmann 45	<0.60	0.74
R. Front. lobe, SFG	0.71	<0.60
R. Thalamus, VAN	0.64	<0.60
R. Front. lobe, Precentral Gyrus	<0.60	0.70
R. Front. Lobe, IFG Brodmann 44	<0.60	0.71
Cuneus	0.70	<0.60
L. Occip. Lobe, Ceneus Brodmann 19	0.65	<0.60
L. Temp. Lobe, SOG	0.66	<0.60
R. Front. Lobe, IFG, Brodmann 9	<0.60	0.63
L. Occip. Lobe, SOG Brodmann 39	<0.60	0.64

These values represent the absolute Pearson correlation between different Talairach regions and region #41 (threshold to above 0.6). Twenty-six regions show a Pearson correlation with region #41 above 0.6 for anxious children, but only 16 for non-anxious children. *STG* Superior temporal gyrus, *ITG* Inferior temporal gyrus, *MTG* Medial temporal gyrus, *WMT* White matter tract, *IFG* Inferior frontal gyrus, *VAN* Ventral anterior nucleus, *SOG* Superior occipital gyrus, *SFG* Superior frontal gyrus.

### Negative stimuli classification

#### Model classification of fearful versus angry faces

**Figure 6:**
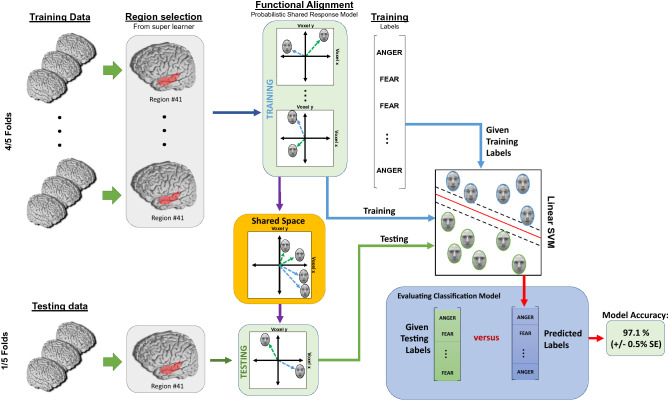
Posthoc analysis: Preprocessing pipeline for negative stimuli analysis. Voxels from the selected region (from the primary analysis) were used to predict the stimulus label for each time point (fear versus anger). We used a probabilistic shared response model (SRM) to transform all functional images into a shared common space. Thereafter, a linear SVM was trained on the functionally aligned data, which were used to predict a facial stimulus for each time point, for each subject. Model metrics include mean accuracy and standard error from fivefold CV.

**Figure 7:**
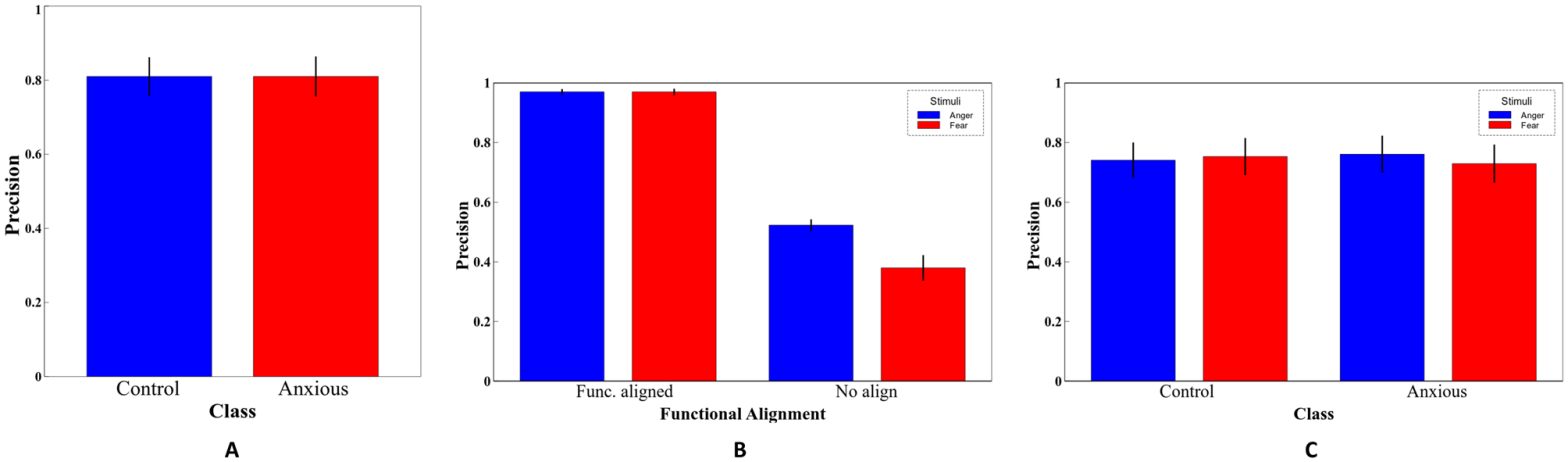
Precision tables for both anxious versus non-anxious and negative stimuli classification analysis. (**A**) Precision table for anxious versus non-anxious AdaBoost classification model. (**B**) Precision table for fearful versus angry faces, linear SVM with and without functional alignment. (**C**) Precision table for four-class classification model of negative stimuli and disease state. All error bars represent the standard error of the precision across outer-CV folds.

As a posthoc analysis, we trained a linear Support Vector Machine (SVM) to predict whether a participant (either anxious or not) was viewing a fearful or angry face at a given time, using only voxels from region $\#41$, which was predetermined from our SL. We applied a probabilistic shared response model (SRM) to functionally align the shared feature space across all subjects, as seen in Fig. 6. Using 5 fold-CV, with this new representational space, we achieved an accuracy of 97.1% (STE ± 0.43%) with a precision of 97.5%, a recall of 97.1%, and an F1 score of 97.1% when classifying fearful and angry faces. Please refer to Table S2 for evaluation metrics. When we trained the same model without functional alignment, we only achieved an accuracy of 49.4% (STE ± 0.81%), a precision of 45.1%, a mean recall of 49.4%, and an F1 score of 42.7% as seen in Table S2. Figure 7B reveals the precision of the SVM with and without functional alignment.

#### Four-class classification

This final classification model coupled the predictions from our primary analysis and negative stimuli classification models into a four-class performance task. For each time point, our ensemble model predicts which group the subject was from (anxious versus non-anxious), and what type of stimuli he/she was viewing at that time (anger versus fearful faces). Our model was able to achieve a balanced accuracy of 73% (STE ± 0.06%) which is an improvement from baseline (26%). Mean precision, recall, and F1 scores were also 73%. Non-anxious children viewing angry faces revealed the highest recall at 76%, and non-anxious children viewing fearful faces revealed the highest precision at 75% as seen in Fig. 7C and Table S3. No significant differences were found between the 4 classes with respect to their precision, recall, or F1 scores.

## Discussion

This study illustrated that a data-driven, machine learning approach can be used to distinguish anxious children from non-anxious children and identify which regions may be important for this performance task. Talairach region $\#41$ (aka, Brodmann’s area 38, right temporal pole, planum polare, or area TG) can be used to distinguish anxious children from non-anxious children based on their brain scans, as they view negative facial stimuli. We demonstrate that task-based fMRI activity related to this anatomical area is sufficient to achieve a relatively high accuracy. In our primary analysis, we trained an SL to parcellate regions and train a non-linear model on those regions. Region $\#41$ was selected as part of our final model, which had an accuracy of 81%. This was compared to a model which included whole-brain activity (54% accuracy), and a searchlight analysis (59.2% accuracy). When examining confounding variables such as age, anxious subtypes and scanner sites, they did not seem to drive the accuracy of our model. Though, we could not run statistical tests because the subtypes and age groups samples were too small in some cases. It is difficult to discern whether our model is partial to certain subtypes of anxiety, but our results suggest the SL performed well regardless. Additionally, we examined the functional connectivity between region $\#41$ and other areas and found that anxious children showed similar correlational patterns between several regions that make up the affective network. In addition, anxious children also exhibited more and stronger correlational patterns to other brain regions compared to non-anxious children. These patterns seem to be part of a distributed neuronal network related to mood regulation and affective processing^27,33^. However, very little research has been conducted on this particular region in relation to pediatric anxiety.

In our posthoc analysis, we examined how neural signatures differed between fearful and angry faces in both anxious and non-anxious children for region #41. When distinguishing neural activity between fearful and angry faces, we were able to achieve an accuracy of 97% with a linear SVM, but only after applying functional alignment (probabilistic SRM) to the brain scans of all children. This suggests that fearful and angry faces are highly dissociable when projected onto a common shared space. Functional alignment can provide enhanced predictive power because it automatically reduces the feature space, while aligning the vectors between subjects to a shared common representational space^25^. We then trained a linear model to make individual predictions from both of our previous models in a four-class classification task that predicted the disease state and the type of facial stimuli simultaneously. Here, we achieved an accuracy of 73%, suggesting we can identify both neural signatures of anxious children and how they process fear and angry faces.

Due to the diverse structure and connectivity to a number of regions, the putative role of the TP has been inconsistent and subject to significant debate^10,34,35^. The TP has been proposed as a social-emotional cognition hub that receives various sensory inputs from limbic structures to organize social processes^34^. Emotional facial processing is a particular type of social process, and young children suffering from anxiety seem to show functional dysregulation in related key limbic structures such as the amygdala and the PFC^15,19^. Our results show that the TP also plays a crucial role in facial processing in children. Using only the neural correlates in the TP, we were able to make individualized predictions about which children suffered from anxiety using a non-linear machine learning model. This suggests that altered functionality exists in this region during a facial processing task involving negative or threat provoking stimuli. This is a novel finding in relation to pediatric cases of anxiety.

### Neuroanatomy of the temporal pole (TP)

The TP lies between the O-PFC and the amygdala^10^, sitting near the anterior end of the temporal lobe, rostral to the perirhinal cortex. It has significant neural connections with the amygdala and PFC via the uncinate fasciculus, making it a paralimbic region^10,36^. Although it is known for processing language, functionality surrounding the TP has also been linked to facial, emotional, and social processing, but it still remains largely understudied^37^. Below, we present findings in neuroanatomical studies in macaque monkeys, showing patterns of connectivity similar to those exhibited in the human brain^38–40^. Additionally, the TP is anatomically close to (and highly connected to) other areas related to facial and socioemotional processing^10^.

### Evidence of socioemotional processing in the temporal pole

Using data from macaque monkeys, Kondo, Saleem & Price (2005) hypothesized that the TP modulates emotional functions related to salient perceptual stimuli based on anatomical connectivity^41,42^. The ventral region receives input from visual processing centers and is considered to be an endpoint in visual processing in macaques^41^. Neurons in this region respond to complex stimuli and change in activity related to visual memory tasks^38^. In humans, neuroimaging tasks have shown that neurons in the TP also responds to complex visual stimuli such as faces. Studies of visually evoked negative emotions, such as fear and anger, have observed changes in activity of the right-ventral region of TP^43,44^. Right-lateralized regions of TP have been implicated in high-level sensory representations with emotional and social experiences, while left-lateralized regions have been associated with linking semantic memory to high-level representations such as faces^10^. Specifically, damaged left TP studies revealed deficits in proper naming abilities and face-name associative learning tasks^45,46^. Additionally, epileptic damage to the right TP has resulted in higher prevalence of anxiety and depression disorders compared to the left TP^45^. Here, it is evident that the TP plays a role in processing emotionally balanced facial expressions. In childhood anxiety, this cognitive process is compromised. Research remains focused largely on amygdalocentric systems, but our results suggest that the activity of right TP alone enables distinctions between anxious and non-anxious children.

As outlined above, research into neural aspects of socioemotional processing has focused mainly on areas such as the amygdala and the prefrontal cortex, not the TP. It has not received the same attention as the amygdala and PFC in emotion studies^10^. In addition, the awkward anatomical placement of TP near the air-tissue boundaries of the sinuses is associated with weaker BOLD signals, making it difficult to draw consistent and statistical significance from fMRI measures^47^. Nevertheless, in our study, the right TP revealed neural differences in socioemotional processing of facial stimuli in anxious and non-anxious children.

### Implications in childhood anxiety

Since 2000, a number of neuroimaging studies have found deficiencies in facial-emotion recognition among individuals suffering from anxiety. In most cases, research has focused on two brain regions—amygdala and PFC—when examining distinct functional differences in anxious individuals while viewing emotional faces^45–48^. Cognitive schema theories suggest that negative or threatening faces receive preferential and early processing advantages through these parts of the brain. It is known that rapid, direct processing of rudimentary sensory stimuli from the thalamus can reach the amygdala in short succession^49,50^, and the amygdala also receives sensory input from indirect pathways such as the PFC, where it assigns significance to the sensory stimuli based on context and prior experiences. This pathway reaches the amygdala in a slower fashion, but conveys higher-order representations and is relevant for memory consolidation^51^. Top-down modulation of this pathway may exert inhibitory influences on the amygdala. In adolescent anxiety, modulatory pathways may become dysregulated, allowing the amygdala to become hyperactive. Numerous studies have cited the PFC-amygdala network in emotional facial processing tasks^47,49,52,53^. While this area is relatively well-known to be implicated in childhood anxiety, it is not the only area where functional differences may be observed.

In trying to conceptualize the functional relationship between anxiety and facial emotional processing, the TP should be considered. American neuroscientist Joeseph LeDoux argued the notion that the amygdala is the fear center of the brain. Instead, he posited that conscious fear is a cognitively assembled experience, derived from many other brain regions, which is not to be confused with the amygdalocentric, non-conscious process of detecting and responding to threats^54^. Additionally, several theories on emotional processing have moved away from the notion that the amygdala is part of a dedicated and prioritized network for assigning emotional valance to ecologically salient stimuli^55^. Revised hypotheses posit that the amygdala is a modulatory center with wide-ranging networks to other brain areas. Thus, it may be responsible for processing information related to salience, significance, ambiguity and uncertainty, assigning biological value to external stimuli^55–57^. Others have cited the amygdala as part of a “whole-brain phenomena” for constructed emotions^58^. One author postulated that the dynamics of the amygdala is used to make predictions of the external world rather than react to it. This is done in the service of allostasis (an organisms’ attempt to efficiently ensure resources for physiological systems in order to survive and reproduce)^58,59^. Emotions serve as constructions or predictions of the external world. They are part of an integrated system that mobilizes the brain and the body to ensure allostasis is maintained. Thus, the amygdalocentric view of emotional regulation should be revised to include a wider array of brain regions, such as the TP.

Few studies have focused on paralimbic regions such as the TP and its connectivity to the amygdala, perhaps due to its later, high-level processing onset of complex stimuli. As mentioned earlier, the TP is (1) heavily connected with the amygdala, (2) responsible for processing complex visual and auditory stimuli, and (3) has been shown to integrate social and emotional significance to said stimuli. Together, these areas are a part of a larger-scale, resting-state brain network known as the affective network^33^. This network has been implicated in various anxiety disorders where individuals are characterized by hyper-arousal, heightened worry states, increased sensory processing and poor emotional regulation^28^. Our connectivity analysis provides strong evidence that the affective network is at play in adolescent children. Not only was the TP a strong predictor of childhood anxiety, but it also showed strong correlational patterns with other affective network regions such as the amygdala, STG, the orbital frontal cortex (OFC), and the border of the insula (Brodmann area #45) as seen in Fig. 5 (right) and Table 2. In addition, anxious individuals exhibited strong correlations for neural activity between the TP and visual cortical areas such as the occipital cortex and the fusiform gyrus. These regions have been linked to the perception of emotion in facial stimuli^60^. Although the affective network domain is based on resting state brain paradigms, similar regions in our task based study were identified by our model. Regions within the affective network seem to show the greatest discriminative power between anxious and non-anxious children. This only reinforces the notion that the TP must be examined in more detail, as it is part of a larger affective network that regulates emotional and perceptual stimuli.

Various brain studies have focused on functional and structural differences in the bilateral TP between different populations during emotional stimulus tasks. While none have emulated the paradigm we have presented, certain parallels exist to confirm our findings. From a social and emotional standpoint, some fMRI and PET studies have shown activation of the TP in such tasks. Bilateral activation of the TP has been seen in negatively valenced films inducing sadness^61,62^, and right TP activation has also been noted in viewing sad and angry faces^63^. Recalling past anxious and angry experiences have shown bilateral activation as well^64^. This suggests that emotionally valenced stimuli do operate within the TP. In our study, we applied functional alignment techniques to better distinguish between various emotional stimuli such as fear and anger in anxious and non-anxious children. Our goal here was to further examine whether types of emotional stimuli differ in their neural signatures between groups in the TP. Using only this region, we were able to distinguish diagnostic labels using the emotional stimuli of fearful and angry faces. This implies that anxious children process fearful and angry faces differently from each other, and they also process these emotions differently from non-anxious children.

While few studies have been conducted on children with anxiety, there are a few sources that cite the TP. A study focusing on group-level differences between SP and GAD in young adults with SP showed increased BOLD activity in the TP and the amygdala when responding to fearful faces compared to young adults with GAD and the control group^65^. The GAD group showed increased activity for angry faces in Brodmann area #10 (which includes part of the PFC) and the middle frontal gyrus, but showed a decrease in activity for the amygdala compared to SP and control groups. The authors concluded that the amygdala was not a sufficient area alone to distinguish group and stimulus level differences in young adults with SP and GAD, recommending that other areas be examined as well^65^. A meta-analysis on SAD in adults revealed abberant activity in affective and default mode network regions such as the right TP, insula, PFC and the precuneus. The authors provided evidence that cognitive processes such as self-referential processing and Theory of mind are linked to these brain regions in SAD adults^66^. Lastly, a resting-state MRI study that analyzed the functional connectivity between the amygdala and the TP in GAD patients revealed some interesting caveats^67^. Compared to the control group, GAD patients revealed an increase in functional connectivity between these regions. They contend that this altered connection may contribute to the etiology of GAD in older patients. Our study confirms the same findings, except for children. Based on our connectome analysis, anxious children had a higher correlation between the right TP and the amygdala as well as the PFC, while non-anxious children did not. This may reveal that innervation’s between the TP and other limbic regions may manifest in young children, and remain into adulthood.

One looming question that remains is the progression of anxiety and its relation to TP as an individual enters adulthood. Specifically, how does the TP affect social and cognitive abilities for a child entering adulthood with clinical anxiety? One premise to consider is Theory of mind and how dysregulated socioemotional processing can result in a failure to respond adequately to social interactions^10^. The inability to properly assess emotional faces and information of others could result in reduced positive and rewarding emotions after a social interaction, leading to maladaptive behavior^68^. A review on SAD in adults showed strong correlations between abberant self-referential processing, Theory of mind and subsequent dysfunction in sub-cortical brain regions such as the TP^66^. Additionally, adults with damage to the right TP have exhibited introversion and coldness, perhaps due to the failure to derive pleasure from social interactions^69^. Reinforced behavior to partake in socialization may be reduced and persist into adulthood^70–72^. This trend has been seen in disorders such as Attention-deficit hyperactivity disorder (ADHD) and autism (ASD)^73,74^. In non-clinical groups, children who benefit from accurate cognitive reappraisal and theory of mind go on to show linear, or even quadratic increases in activity in areas such as the right TP as they move to adulthood^68^. While it is difficult to ascertain if the TP directly causes socioemotional dysregulation into adulthood for anxiety, the fallout from poor theory of mind may persist into adulthood. Anxiety disorders are among the most persistent mental health disorders propagating from adolescence to adulthood, with a core criteria of symptoms centered around social and emotional processing^75^. It may be that dysregulation of the right TP in childhood anxiety may proliferate into adulthood.

Currently, there is no validation or diagnostic procedure that involves any component other than clinical signs and symptoms via psychiatric assessment. Although neuroendocrine, cognitive, genetic, and neuroanatomical correlates exist, there is no available biological test for diagnosis. Here, we used a facial processing fMRI task to not only classify anxious from non-anxious children, but to also distinguish between the affective stimuli presented. Instead of using a multi-variate strategy to examine whole-brain neural patterns, we focused on one particular region, which has been implicated but understudied in anxiety and socio-emotional processing. The TP served as an anatomical region that could predict which children suffered from anxiety based on the neural correlates of fearful and angry faces.

### Limitations and future work

One future consideration is to conduct a meta-analysis to other studies of anxious children or adults. As mentioned, there are few papers focusing on the temporal pole as a region that could be implicated in anxiety. If data is available, validating our model on adult cases could yield interesting findings. Another consideration is to test our model on a separate cohort dataset with more subjects. Ideally, evaluating the performance of our proposed approach could benefit from a more homogeneous target group. Since our target group contained children with 3 different types of anxiety disorders (co-morbid disorders as well), the variance between the neural signatures of these children may have differed significantly. There is evidence that SP, GAD, and SAD show different neurological and behavioral patterns between each other^65,76,77^. Thus, confounding effects may exist within the anxious group that could affect the overall performance accuracy. However, comparing SP, GAD, and SAD is out of the scope of this paper. Another limitation may be that functional alignment could result in high accuracies in other areas than the TP for our secondary analysis. The TP was critical for the primary analysis classification, but was comparable to other brain regions in the secondary analysis after functional alignment was applied. Another future consideration could involve transfer learning, a machine learning technique that involves training with one type of labeled data (﻿i.e., train a model using only GAD participants), then applying that model on other classes (SP or SAD children) to examine whether the model can correctly distinguish cases based on the prior knowledge of only GAD children. Another potential limitation was the absence of other facial stimuli in the task. Carpenter et al., (2015) only released functional scans with fearful and angry faces for the purposes of their study^77^. Thus, we had to focus on negative stimuli only, and although we successfully distinguished fear from angry faces in the brain, other facial stimuli may offer further insights into how emotion is processed in the brain of anxious and non-anxious children.

## Conclusion

In summary, the goal of this study was to use a data-driven approach to classify anxious versus non-anxious children using emotional facial stimuli. Here, we used a super learner (AdaBoost with logistic regression as a base estimator) to select the Talairach regions that could best distinguish anxious from non-anxious children. Our model achieved an accuracy above 81% for this task. Subsequently, we examined how different negative emotional faces would be processed in both groups. We found that fear and angry faces could clearly be distinguished in the TP, but only after functional alignment was applied to the brain scans of all subjects. This study illustrates the power of task-based fMRI designs to predict disease states and stimulus conditions. It also indicates that the TP is a region that should be further examined in pediatric anxiety. Cognitive processes such as emotional facial processing may be compromised in anxious children. We have demonstrated that machine learning analysis of face-processing, task-related fMRI data may be used to distinguish anxious from non-anxious children. This may enable further understanding of neural underpinnings of pediatric anxiety and help to extend and validate diagnostic labels used by psychiatrists and other clinicians.

## Methods

### Data and code

This paper analyzed data provided by Carpenter, K.L., Angold, A., Chen, N.K., Copeland, W.E., Gaur, P., Pelphrey, K., Song, A.W. and Egger, H.L. (2015), who posted their data-set on https://openneuro.org. The link to the data repository is https://openneuro.org/datasets/ds000144. Their data was made publicly available on 2018-03-26^77,78^. All analysis can be replicated using our GUI-based toolbox, easy fMRI. The GitLab repository can be found and cloned at https://easyfmri.learningbymachine.com/.

### Recruitment

Secondary analysis of existing data was obtained from Carpenter et al., (2015). Children were initially recruited from the Duke Preschool Anxiety Study (DPAS), which was a longitudinal, multi-phase study. The last phase was entitled “Learning about the Developing Brain study” (LABD), where 208 children who participated in previous phases of the DPAS were recruited to take part in this study, which examined brain development in children suffering from anxiety. Of the 208 children, 155 were eligible to participate in the neuroimaging phase. Children who met the criteria for generalized anxiety disorder, SP, and/or SAD were recruited into the “case” group, and children who did not meet the criteria for an anxiety disorder were recruited as the comparison group. Children in the LABD were not excluded for comorbid non-anxiety disorders or for taking psychotropic medications^77^.

Parents completed the Preschool Age Psychiatric Assessment (PAPA) for children involved in this study^79^. The PAPA is a diagnostic instrument for assessing psychopathology of children aged 2–9, and it is based on the parent version of the Child and Adolescent Psychiatric Assessment^77,80^. Frequency, duration, and the onset of symptoms are collected to determine whether the child meets the diagnostic criteria for anxiety disorders in the Diagnostic and Statistical Manual of Mental Disorders (DSM-IV). The PAPA assesses symptom severity during the previous 3 months, as shorter recall periods have been shown to reflect more accurate recall^80^. A composite score of GAD, SP, SAD, and depression symptoms were obtained from the PAPA and was used as a measure of school-age emotional symptomatology^77^.

This study was approved by the Duke University Medical Center Institutional Review Board, and was carried out in accordance to U.S regulatory requirements related to the protection of human research participants, which include the Accreditation of Human Research Protection Program (AAHRPP) and the Health Insurance Portability and Accountability Act (HIPAA) guidelines. Verbal assent from the child and informed consent from the parent were obtained after a full description of the study was presented. Children and parents were financially compensated with gifts. or money vouchers^77^.

### Participants

Children eligible for the fMRI study had to meet 3 requirements: (1) They completed the first phase of the DPAS study, (2) they must be older than 5 and half years old, (3) have successfully completed a mock scan session in the MRI machine. Children were placed into one of two groups. The first group involved anxious children, who met the criteria for GAD, SP SD, or some combination of the 3 using the PAPA questionnaire, and non-anxious children who served as a control group. Of the 155 children initially recruited, only 45 had usable data due to a number of reasons including parents or child refusal to take part, absentees, excessive motion in the scanner, and lower IQs^77^. Of those 45 children, 22 were in the anxious group and 23 were in the non-anxious group. The anxious group contained individuals with either one or more anxiety disorders. Within the anxious group, 15 children met the criteria for GAD, 11 for SP, and 10 for SAD. 12 out of 22 anxious children met the criteria for more than one anxiety disorder. The age range of both groups was between 5.5–9.5 years old, as seen in Table 1. Impairment and emotional symptoms were recorded prior to the start of the fMRI study and were representative of psychiatric symptoms that interfere with daily functioning. Impairment scores were assessed using the World Health Organization’s International Classification of Functioning, Disability, and Health^81^. Emotional symptoms were measured on a composite scale that accounted for both anxiety and depressive symptoms^77^.

### Functional MRI task

The fMRI task was a block design, emotion face processing task. Facial stimuli from the NimStim Stimulus Set were selected (45), but only angry and fearful faces were used according to Carpenter et al., (2015). Each subject completed 2 blocks. At the beginning and end of each run, there was a 16-s fixation block and 15-s task blocks were stationed in between and separated by 12-s baseline fixation blocks, which consisted of a colored star in the center of the screen. Faces were shown for 1.25 s with no inter-stimulus interval. Each run contained 3 blocks of fearful and angry faces exclusively, with the order of the emotional faces randomized. To make sure the children were staying engaged, they were instructed to press a button whenever a face with glasses was shown on screen. These faces were randomly placed throughout the blocks and expressed the same emotion as the other pictures within the block. The average task accuracy was 83.33% for non-anxious children and 82.29% for anxious children. The study used a block-design fMRI scheme in which all participants viewed the same number of stimulus presentations and consistent interstimulus intervals. This experiment scheme allowed us to extract 35 time points (based on our design matrix) for each participant during preprocessing. Further, these time points were also temporally aligned to ensure m$^{{\mathrm{th}}}$ time point for all participants represented the same type of stimuli.

### MRI acquisition

MRI acquisition was completed on two different 3T GE scanners. Of the 45 participants, 15 (8 anxious, 7 non-anxious) were scanned using the EXCITE HD system, and 30 participants (14 anxious, 16 non-anxious) were scanned on the MR750 system. Parameters and pulse sequences were congruent between the two systems, and calibration metrics such as spatial accuracy and dynamic signal stability were validated using an agar phantom (soft tissue mimic). In both systems, scans lasted 5 minutes and 44 seconds and 172 functional images were generated during the task. For each run, between 34–39 slices were generated which were parallel to the AC-PC plane using a BOLD-sensitive EPI sequence (voxel size: 4 mm$^{3}$; Repetition time: 2000ms; Echo time: 27ms; Field-of-view: 24 cm; Flip-angle: 77; Interleaved-odd acquisition)^77^. Co-registering the functional images was done in conjunction with a high resolution T1-weighted anatomical scan using the 3D-FSPGR sequences with SENSE (voxel size: 1 mm$^{3}$; Repetition time: 8.096ms; Echo time: 3.18ms; Inversion time: 450ms; Field-of-view: 25.6 cm; Interleaved-odd acquisition)^77^. Batch effects were recorded as a covariate in the machine learning analysis to ensure manufacturing differences between the two systems were not a cause of functional differences.

### Pre-processing

Data was pre-processed and analyzed using Easy fMRI (version 1.8B8800) (https://easyfmri.github.io) and FMRIB Software Library (FSL version 6.0.3). We have used the “fMRIPrep” pipeline^82,83^—which includes brain extraction, registration to standard space, motion correction, slice time correction, normalization, and spatial smoothing. To prepare the images for registration, we first used the Brain Extraction Tool (BET) to eliminate non-brain tissues such as the scalp and brain marrow. We then registered all the subject’s brain images to a common reference coordinate system using the MNI-152, 2 mm resolution (T1 weighted) standard space. To anatomically align the brain images, we used an affine (12 degrees of freedom, 12 DOF) transformation to rotate, translate, and scale the images into alignment^84^. Motion correction was also handled in this affine transformation. Due to movement in the scanner, we needed each voxel to correspond to a consistent anatomical point for each point in time^84,85^. Here, we chose to use the first image in the time frame to reference all other volumes at other time points. Fortunately, the dataset we acquired already removed excess motion subjects. Carpenter et al., (2015) removed relative and absolute motion and intensity jumps greater than three standard deviations from the run mean as part of their scrubbing protocol. The mean of runs was determined by taking the absolute deviation relative to the mean of runs after each voxel was passed through a high pass filter to remove low-frequency drifts (1/60 Hz)^77^. Task blocks were removed from analysis if two volumes were removed from the start of the block or more than 3 volumes in total were removed from the block. Additionally, the entire run was excluded from subsequent analyses if more than one block of emotional stimuli was removed^77^. Next was spatial smoothing. Spatial smoothing is a method used to increase the signal-to-noise ratio in fMRI brain volumes. Smoothing was done by using a 3D convolution with a Gaussian kernel to replace voxel intensities with a weighted average of neighboring intensities. We specified our Full-Width-Half-Maximum (FWHM) kernel to be 5.0 mm. After, we applied a global intensity normalization between subjects and sessions. Lastly, we used temporal filtering, which is a removal of high and or low frequencies in the raw signal of voxel intensities via band-pass filters. In a time series of each voxel, there may be scanner related or physiological signals that cause high-frequency noise.

### Analysis: Anxious versus non-anxious classification

#### Machine learning analysis

Using the Talairach atlas (with 2mm voxel size), we used a super learner (SL) that segmented brain regions (959) and used them as hyper-parameters to examine which areas could best separate our diagnostic labels. Figure 1 illustrates the full machine learning pipeline for our primary analysis. All machine learning analysis was done using Python 3.9^86^ or in Easy fMRI^83^. Subsequent libraries included scikit-learn (version 0.23.1)^87^, SciPy (version 1.6.1)^88^, Pandas^89^, and Numpy (version 1.20.1)^90^.

A SL is seen as an “ensemble of ensembles” that combines models or model configurations on the same split of data, and then uses out-of-fold predictions to select the best configurations or models^91^. We applied the whole-brain data to a SL—where it parcellated the neural responses based on the Talairach atlas and then found an optimal prediction model for each of the regions. The SL returns the model which can best distinguish class labels (anxious versus non-anxious). The SL would make a prediction on every time point for each subject (35 time points), then use a majority vote to make a final prediction for the class label associated with that individual, regardless of task stimuli. We split the data using fivefold CV based on the participant IDs (36 / 45 participants were considered our training set, and 9 / 45 our testing set). The SL used a nested cross-validation process, whereby the outer CV process used 4/5$^{\mathrm{ths}}$ of the participants for a training set (balanced for diagnostic labels), and 1/5$^{\mathrm{th}}$ for a testing set. Within the training set, another 5 fold-CV was used to fine tune the hyper-parameters within the SL. Folds were split based on the participant ID instead of time points (30 / 36 participants were in the training set, and 6 / 36 were in the validation set). An ensemble classifier (AdaBoost) with a logistic regression base estimator was used to make the predictions on each participant.

AdaBoost (short for Adaptive Boosting) is an ensemble machine learning paradigm where multiple models (often called “weak learners”) are amalgamated in such a way to achieve more robust results^91^. This is done by setting weights for both the weak learners and the data points. The algorithm forces the weak learners to concentrate on observations that are difficult to classify correctly. AdaBoost uses boosting, a sequential ensemble process that gives misclassified cases a heavier weight, samples without replacement, and reduces the bias-variance tradeoff by combining weak or shallow learners together in a voting process to make predictions^91^. We used four hyper-parameters to tune our classifier within the inner CV. Hyper parameter tuning was done using GridSearchCV, a function within Scikit-learn^87^. First, we used a different number of estimators [n_estimators = 10, 50, 100, 150] to determine the maximum number of estimators at which boosting is terminated. Second, we adjusted the learning rate [learning_rate = 0.05, 1, 2]. Third, we changed the number of max iterations completed by AdaBoost [max_iterations = 100, 500, 1000]. Next, we tuned the type of regularization performed by the logistic regression estimator [penalty = L1, L2, none]. This was also coupled with the regularization penalty variable [C = 0.5, 1, 2]. Lastly, we used the segmented regions from the Talariach atlas as a hyper-parameter. We evaluated the performance of our results by using the accuracy, which was computed as the average accuracy across the folds in the outer CV. We used precision, recall and F1-score to evaluate the final model.

#### Statistical analysis of top region(s)

We conducted a high level, between-group analysis for the ROI selected to examine activation differences for anxious versus non-anxious children. Instead of using a regular classification analysis, we conducted a grouped Bayesian representational similarity analysis (GBRSA) that can compare the (dis)similarities between different cognitive states across multiple participants. This was done to determine whether the pattern of activity between anxious and non-anxious children were statistically different in region #41.

RSA is a similarity fMRI analysis method that explores the neural response patterns of brain regions across different stimuli or different groups^92,93^. Using a measure of similarity or dissimilarity (1-measure) such as euclidean distance, Spearman’s correlation or Pearson’s r, neural activity regarding stimuli can be compared to each other, resulting in a representational (dis)similarity matrix (RSM or RDM)^93^. From there, non-parametric statistical tests can be conducted to compare neural activity across stimuli, groups or both^92^. In our case, we used a between-group analysis of anxious and non-anxious children, regardless of what stimuli they examined.

Traditional RSA has been widely adopted in cognitive neuroscience, but suffers from some confounding factors. Mainly, similarity metrics tend to be much higher when neural patterns are in close temporal proximity, which can conflate results^94^. Secondly, traditional RSA can result in unstable (bias) analysis when the signal-to-noise ratio is low for some sets of data^94,95^. GBRSA is a Bayesian extension of RSA that can address the mentioned issues. While RSA uses deterministic approaches (e.g., general linear model or ordinary least squares) to estimate the similarity between the neural responses, GBRSA uses the maximum likelihood estimation (MLE) to learn hyper-parameters of a distribution for the neural responses of each subject—while a single covariance matrix is used across all subjects to maximize the joint probability of observing neural responses. we use a shared covariance matrix^95^. So, GBRSA improves on these issues by reducing the temporal and covariance bias—i.e., learning the covariance structure as a hyper-parameter. By reducing the unknown activity patterns across anxious and non-anxious children, a direct estimation can be made from the covariance matrix^94^. Once generated, we measured neural activity in the TP to determine whether either group differed from each other using a Mann-Whitney U-test, which does not assume our neural activity has a normal distribution. Analysis for GBRSA was done in Easy fMRI and Python^86^, which included the library SciPy to conduct the Mann-Whitney U-test^88^. Brain images seen in Fig. 5 were done using Analysis of Functional NeuroImages (AFNI_21.1.01), Surface Mapping (SUMA) and Easy fMRI^83,96,97^.

#### Fully connected network analysis

Lastly, we wanted to look at a fully connected network analysis between the highest selected region and all other regions. To do this, we first partitioned the raw neural activities between anxious and non-anxious children. Next, neural activities were further partitioned based on 959 regions of the Talairach atlas. We then averaged the neural activities within each Talairach region across all voxels—which resulted in a vector with the same size as our time points. After, we compared each of these vectors by calculating the absolute value of the correlation in a similarity matrix. We then applied a threshold to examine the most correlated regions (top 30%). Finally, we visualized both of the anxious and non-anxious networks and only showed the top connections with our highest selected region. This was done to examine whether our ROI showed different neural connections to different areas of the brain in anxious and non-anxious children, regardless of facial stimuli.

### Analysis: Negative facial stimuli

#### Model classification of fearful versus angry faces

We also sought to distinguish fearful versus angry faces among the neural activity of all children with our ROI. For between-subject comparisons, tasks such as pattern classification or RSA yield lower accuracies because the representational spaces are highly dimensional, the functional topography may be different between subjects and anatomical brain structures vary between participants. Thus, a recent method known as functional alignment has been proposed to align patterns of neural responses across subjects into a common, lower-dimensional space^25^. One important assumption is that we assume all human brains have similar neural activity for experiencing the same categorical stimuli. Here, we used a probabilistic shared response model (SRM) to functionally align neural activity for fearful and angry faces for all subjects only in our region of interest^98^.

SRM uses the training data to learn the mappings for each subject’s data shared feature space. Then these learned mappings are projected onto the held-out data for each subject into a shared feature space. One of the main distinctions in SRM is that the model directly estimates that the selected shared features are significantly less than the number of voxels it is selecting from. This is different from other methods, where the number of features usually equals the number of voxels^98^. The machine learning pipeline for our negative stimuli analysis can be seen in Fig. 6. Analysis for SRM was conducted in Easy fMRI.

Once we obtained the functionally aligned dataset for the facial stimuli, we trained a linear SVM classifier on the ROI. Here, instead of using a non-linear model (such as AdaBoost in the primary analysis), we opted for a linear model instead. First, our intuition was that—since functional alignment maps the neural responses for our facial stimuli to a linear feature space, using a non-linear model would increase the chance of over-fitting. Thus, we followed Occam’s razor and opted for a simple, linear model to prevent this issue. Secondly, using a linear model has reduced computational time compared to non-linear models. Fivefold CV was used, but with no internal CV approach this time. Also, no majority vote was used for final predictions. Instead, each time point was individually predicted, and metrics such as accuracy, precision, recall, and F1-score were averaged across each time point between all subjects in the testing folds.

#### Multi-class classification

Taking the final predictions from both our analysis models, we sought to make a four-class classification model that predicts the diagnostic stimuli (anxious versus non-anxious) and the type of stimuli (fearful versus angry faces) in a post-hoc analysis. Using the final predictions from the trained AdaBoost classifier (anxious versus non-anxious) and the linear SVM (fearful versus angry faces), we generated new prediction labels based on the original class and stimuli labels and compared them to the observed labels. A one way ANOVA was conducted to examine differences in precision between the 4 classes. This can be seen in Fig. 7C.

## Supplementary Information

Supplementary Information.
