# Supplementary material for: Predicting pediatric anxiety from the temporal pole using neural responses to emotional faces
Source: Sci Rep. 2021 Aug 18;11:16723. doi: 10.1038/s41598-021-95987-4 (PMC8373898; doi:10.1038/s41598-021-95987-4)
Supplement: Supplementary file 1 — Supplementary Information. [file 41598_2021_95987_MOESM1_ESM.pdf]

Supplementary Materials

Tables

Table S1. Primary analysis, super learner model results. This table reveals the results from the highest region-based model (region #41) and from our super learner. The standard error on the accuracy is computed across the number of folds in the cross-validation process.

Classifier	Class	Precision	Recall	F1 Score	Accuracy
AdaBoost	Non-anxious	82.0 %	79.0 %	82.0 %	
	Anxious	79.0 %	80.0 %	79.0 %	
	Overall	81.0 %	80.0 %	81.0 %	**81.0% (+/- 1.4%)**

Table S2. Secondary analysis model results. This table reveals the results from the highest region-based model (region #41). Our model achieved a 97.1% accuracy for distinguishing fearful from angry faces in both class groups. The standard error on the accuracy is computed across the number of folds in the cross-validation process. A t-test between the two models revealed a non-significant difference ($t_{(21)}$= 0.81, p > 0.05).

Classifier	Class	Precision	Recall	F1 Score	Accuracy
Linear SVM	Fear	97.9%	97.1%	97.0%	
	Angry	97.1%	97.0%	97.1%	
	Overall	97.5%	97.1%	97.1%	**97.1% (+/- 0.5%)**

Table S3. Four-class ensemble classification results. This final classification model coupled the predictions from our group and negative stimuli classification models into a four-class performance task. Our models achieved a balanced accuracy of 73% compared to baseline which was 26%.

Classifier	Class	Precision	Recall	F1 Score	Accuracy
Four-class ensemble model	Non-anxious angry	74.0 %	76.0 %	75.0 %	
	Non-anxious fear	75.0 %	72.0 %	73.0 %	
	Anxious angry	74.0 %	71.0 %	73.0 %	
	Anxious fear	70.0 %	74.0 %	72.0 %	
	Overall	73.0 %	80.0 %	80.0 %	**73.0% (+/- 5.4%)**

Data and Code Availability

The data and code for reproducing results are available in "easy fMRI" (https://easyfmri.learningbymachine.com/). Easy fMRI is a GUI-based environment for applying feature analysis, hyper-alignment, Multi-voxel Pattern Analysis (MVPA), Representational and Similarity Analysis (RSA). Instructions on installing and using easy fMRI can be found at https://gitlab.com/easyfmri/easyfmri. The data for this paper can be found at https://openneuro.org/datasets/ds000144/versions/00002.
